# Case report: Moyamoya disease, the culprit in an adult female presenting with left sided numbness

**DOI:** 10.1097/MD.0000000000032160

**Published:** 2022-12-02

**Authors:** Ohood Kh. Almutairi, Yasser Abdulmoez

**Affiliations:** a Neurology resident, Kuwait Institute for Medical Specializations, Kuwait; b Consultant of Internal Medicine, Internal medicine department, Farwaniya hospital, Kuwait.

**Keywords:** angiography, case report, cerebrovascular disease, HR-VWI, Moyamoya disease, numbness, stroke

## Abstract

Moyamoya disease is a rare progressive cerebrovascular disease that is characterized by stenosis of the vascular branches of the internal carotid artery and the development of abnormal, fragile collateral vessels. Patients with Moyamoya disease mainly present with signs and symptoms of cerebral ischemia or cerebral hemorrhage with a typically progressive course.

**Patient concerns:**

Here, we present a case of an adult female presenting with left sided upper and lower limb numbness.

**Diagnoses:**

Diagnosis is through cerebral angiographic images demonstrating the characteristic look of collateral vessels classically present in Moyamoya disease. In this case, initial angiographic imaging along with high-resolution vessel wall brain magnetic imaging were used to diagnose the patient with this disease.

**Interventions:**

The patient was advised for surgical intervention; however, they were reluctant.

**Outcomes:**

The patient was treated conservatively and advised for follow-up.

**Lessons:**

This case highlights the importance of considering Moyamoya disease in the differential diagnosis of patients presenting with sudden neurological symptoms. High-resolution vessel wall MRI is a useful tool to diagnose this disease.

## 1. Introduction

Moyamoya disease (MMD), first reported in 1957 in Japan, refers to a rare cerebrovascular disease that is characterized by progressive stenosis of the vascular branches of the internal carotid artery; leading to the development of abnormal, fragile collateral vessels. The term Moyamoya was introduced by physicians Suzuki and Takaku in 1969, which means “puff of smoke” in Japanese and it refers to the hazy characteristic appearance of these collateral vessels on angiographic images in patients with this condition.^[1,2]^

MMD is most common in East Asian countries. Its annual incidence is 0.35 to 0.94 per 100,000 population reported in Japanese studies with a slight female-to-male predominance.^[3]^ This disease has been also noticed to have a bimodal age distribution, with peaks occurring at around 10 years and at 30 to 40 years of age.^[3]^

Moyamoya disease mainly presents with either symptoms of cerebral ischemia or cerebral hemorrhage.^[4]^ Though the exact etiology is still not known, about 10% to 15% of patients had a family history of the disease, indicating a possible genetic relationship.^[3]^

Treatment of Moyamoya disease mainly involves surgical revascularization techniques with limited medical treatment.^[5]^

Here, we use angiographic imaging along with high-resolution vessel wall brain magnetic imaging (HR-VWI) to diagnose a 44-year-old female presenting with left sided upper and lower limb numbness with Moyamoya disease.

## 2. Case description

A 44-year-old female presented to the emergency department with left sided upper and lower limb hypesthesia associated with headache, imbalance, and dizziness of 3-day duration.

The headache was diffused, of insidious onset; tension-like, not localized to any area, and not relieved by over-the-counter painkillers. There was no history of nausea or vomiting, loss of consciousness, fever, trauma, speech or vision disturbances, weakness, or any other deficits. The patient had no significant past medical history except for a hysterectomy; done 10 years prior with no complications.

The patient was not on any medications at time of admission. No reported allergies, no alcohol consumption or smoking; and no significant family history.

On arrival to the hospital, the patient was conscious, alert, oriented; her vitals were (blood pressure: 162/110 mm Hg, heart rate: 102/min, temperature: 37°C, respiratory rate: 20/min, and oxygen saturation was 97% on room air, and random blood glucose: 9.22 mmol/L).

On inspection, the patient was well built with Xanthelasma present on both eyelids. She had decreased sensation on the left side of her face compared to the right side. The hypesthesia was equal in all 3 branches of the trigeminal nerve. Otherwise, the cranial nerves were intact and fundoscopy was normal.

The patient also had decreased sensation of the left upper and lower limbs with power of 3/5 compared to the right which had a power of 5/5 with intact sensation. Tone was normal and reflexes were brisk bilaterally. Gait could not be assessed due to severe dizziness. Other systems were unremarkable.

**Figure 1.**
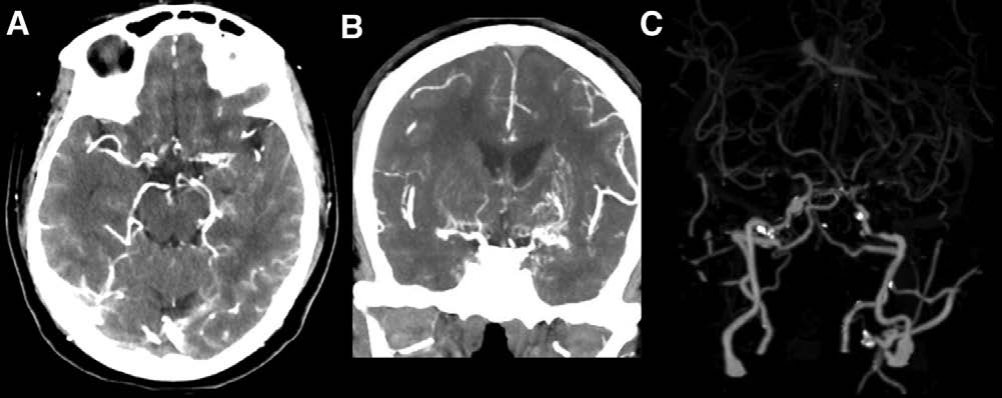
(a) Axial CTA image showing stenosis of the distal internal carotid arteries and M1 segments. (b) Coronal CTA image demonstrating the bilateral thalamic collaterals. (c) MIP CTA image show stenosis of the distal internal carotid arteries. CTA = computed tomography angiography, MIP = maximum intensity projection.

CT angiogram head with contrast was done. Axial (Fig. 1a), coronal, and maximum intensity projection computed tomography angiography (CTA) images (Fig. 1b and c) show stenosis of the distal internal carotid arteries and M1 segments with no contrast filling of the anterior cerebral arteries. Bilateral thalamic collaterals were present and are demonstrated in (Fig. 1b). CT cerebral venography head was unremarkable with normal opacification of the deep veins of the brain.

**Figure 2.**
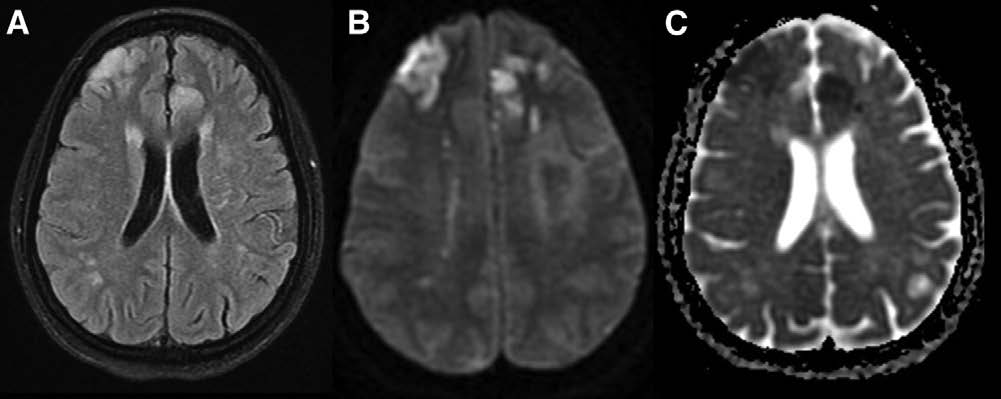
Axial T2 weighted image (T2WI) (a) shows bilateral frontal cortical and subcortical areas of high signal intensity that shows restricted diffusion pattern in diffusion weighted imaging in (b) and low signal on apparent diffusion coefficient (ADC) maps in (c) indicative of acute ischemic insults.

**Figure 3.**
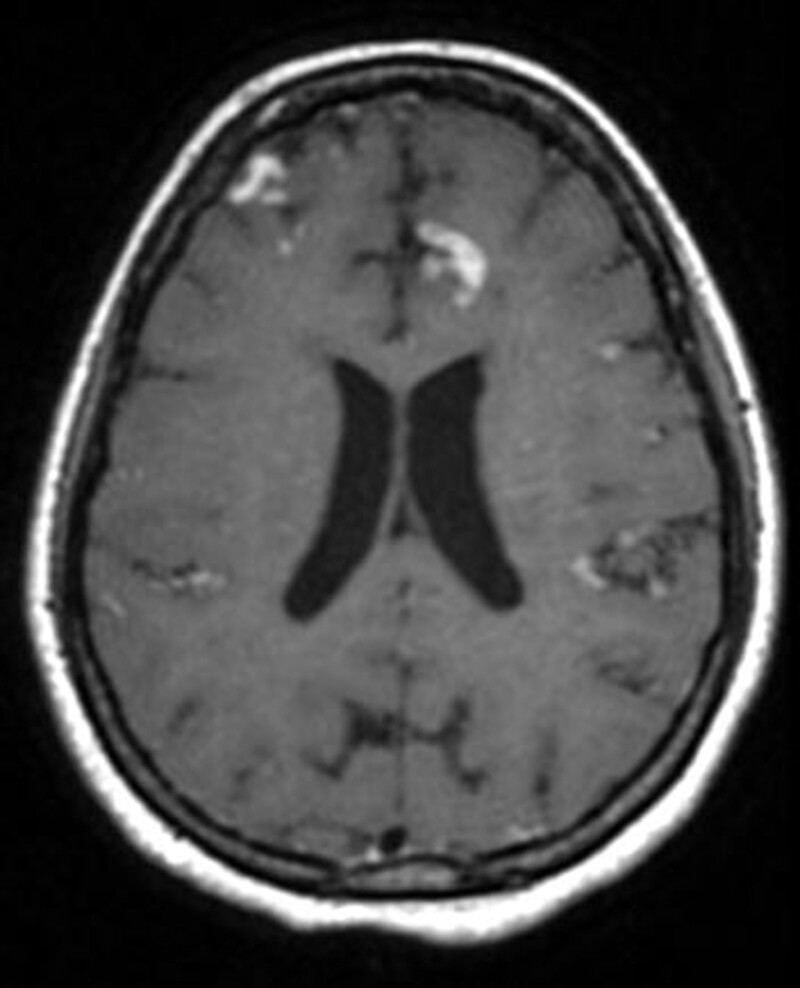
Contrast enhanced T1 images after 5 days interval shows gyral enhancement of the involved areas.

Magnetic resonance (MR) angiography showed bilateral frontal cortical and subcortical areas of high signal intensity indicative of acute ischemic insults (Figs. 2 and 3).

Noting that MR venography was unremarkable.

**Figure 4.**
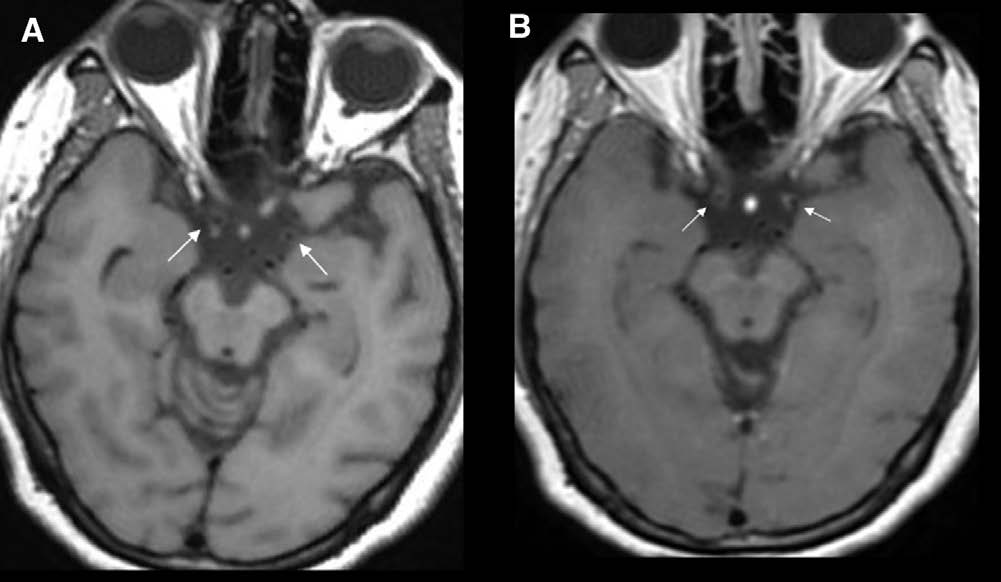
Pre (a) and post (b) contrast enhancement high-resolution vessel wall imaging (HR-VWI) using T1 CUBE shows mural thickening of the terminal portions of internal carotid arterys (ICAs) with poor enhancement (white arrows) as compared to pituitary stalk and normal appearing white matter.

Moreover, high-resolution vessel wall imaging brain MRI (Fig. 4) was also done which showed pre and post-contrast enhancement pattern supporting the diagnosis of Adult onset Moyamoya disease.

Laboratory investigations including complete thrombophilia screen were unremarkable except for the lipid profile which showed dyslipidemia (total cholesterol: 6.23 mmol/L, LDL 5 mmol/L, and triglycerides 1.32 mmol/L).

Other investigations included ultrasound doppler carotid, which showed non-uniform thickening of intima-media complex of the carotid tree bilaterally with evidence of stenotic diameter about 57% at the left internal carotid artery (ICA) and 43% at the right ICA. Echocardiography was normal.

The patient was started on statins, aspirin 81 mg, and appropriate antihypertensives. During her hospital stay, the patient cognition level was deteriorating and slowly started to have expressive aphasia, however, she was able to follow commands correctly. Repeat CT brain showed stationary course of the disease with no new ischemic insults.

The patient was seen by both neurosurgical and vascular surgery specialists, however, the patient’s family refused surgical intervention and insisted on being discharged to travel back to their home country. The patient was discharged on Aspirin 81 mg, statins, and antihypertensives and advised to follow neurosurgery for possible intervention.

## 3. Discussion

Diagnosing Moyamoya disease might impose a challenge and should be guided with clinical presentation and proper diagnostic images. The presentation of this disease differs with 2 age groups; the pediatric population typically present in their first decade of life with progressive symptoms such as ischemic strokes, transient ischemic attacks, seizures, and progressive mental decline. Whereas adults, ages around 30 to 40 years old, more often present with intracranial hemorrhage.^[4]^ Here, our patient presented with ischemic infarcts along with mental decline throughout her hospital stay. The term MMD is used when the cause is unknown or due to genetic susceptibility, whereas, the term Moyamoya syndrome is used when the vascular findings are due to an associated condition, such as sickle cell disease or thalassemia. In this case, the patient was previously healthy and all thrombophilia screen was sent including anti-double-stranded DNA, antiphospholipid antibodies, and rheumatoid factor and all were negative.

Multiple reports with increasing evidence have been published linking Moyamoya disease to the ring finger protein 213 gene. Patients with this gene have an earlier age of onset and more severe disease.^[6–8]^ Unfortunately, genetic testing could not be done in this case due to social reasons.

The diagnosis of Moyamoya disease is made by identifying the unique appearance of bilateral stenosis in the distal internal carotid arteries or proximal portion of the anterior and/or middle cerebral arteries with the characteristic prominent collateral vessels on angiographic imaging.^[9]^

Although conventional digital subtraction angiography is the gold standard for the diagnosis of this disease, noninvasive imaging such as CT angiogram or MR angiogram have a high diagnostic value in Moyamoya disease.

However, HR-VWI is a newer advanced multi-contrast noninvasive technique that has been developed in recent years to allow detailed characterization of the vessel wall greatly improving diagnostic accuracy of several cerebral vasculopathies compared to traditional imaging.^[10,11]^

One study showed that using HR-VWI along with digital subtraction angiography, CTA or MR angiography significantly improved the accuracy of diagnosing Moyamoya disease from 31.6% to 86.8%; and the most common findings were no post-contrast vessel wall enhancement and absence of eccentric wall thickening.^[12]^

In this case, high-resolution vessel wall MR pre and post-contrast (Fig. 4) imaging showed mural thickening of the terminal portions of ICAs with poor enhancement as compared to pituitary stalk and normal appearing white matter supporting the diagnosis of Moyamoya disease.

Treatment of MMD is mainly surgical, including mainly 2 types of revascularization surgeries, termed direct and indirect revascularization.^[5]^ Surgery is done to improve cerebral blood flow and perfusion and to reduce the risk of cerebrovascular complications. Medical treatment in this disease is limited and not well studied. Due to the components of both hemorrhagic and ischemic complications in MMD, the use of anti-coagulation is not recommended due to the higher risk of bleeding and the use of anti-platelet therapy is controversial and should be individualized. A recent retrospective cohort study in an adult population with ischemic MMD, compared the efficacy of surgical intervention, anti-platelet therapy, and conservative management (no medical intervention or the use of other treatments). Surgical intervention was superior as it had the longest interval period between further strokes and it reduced the rate of further cerebral ischemic events (1.9%) compared to the antiplatelet therapy group (5.7%) and the conservative management group (15.1%).^[13]^ A large Korean national retrospective cohort study observed patients with MMD from 2002 to 2016 and it showed strong evidence that the use of antiplatelet therapy decreased mortality by two thirds in those patients compared to non-antiplatelet use.^[14]^

In this case, the radiographic imaging combined with the clinical presentation of the patient supports the diagnosis of Moyamoya disease.

## 4. Conclusion

This case highlights the importance of considering Moyamoya disease in the differential diagnosis of a patient presenting with signs of sudden neurological symptoms. Wall vessel brain MRI can significantly improve diagnostic accuracy of this disease when combined with other imaging like CTA and MR angiography. More research is needed to study further treatment of this progressive disease.

## Acknowledgments

We would like to thank Dr Ali H. Elmokadem, Senior specialist of radiology from the Department of Radiology, Farwaniya Hospital, Kuwait, for his appreciated contribution in providing the description of the radiological images.

## Author contributions

O.A.: is the primary author of this work. Contributed in preparation, creation, and writing of the published work. Y.A.: Critically revised the work. All authors read and approved the final article.

**Conceptualization:** Ohood Kh. Almutairi.

**Data curation:** Ohood Kh. Almutairi.

**Investigation:** Ohood Kh. Almutairi.

**Supervision:** Yasser Abdulmoez.

**Validation:** Ohood Kh. Almutairi, Yasser Abdulmoez.

**Writing – original draft:** Ohood Kh. Almutairi.

**Writing – review & editing:** Ohood Kh. Almutairi.
